# THE BLUES AND OLDER MINORITY MUSICIANS: MORE THAN JUST MUSIC XXVI

**DOI:** 10.1093/geroni/igz038.2891

**Published:** 2019-11-08

**Authors:** John N Migliaccio

**Affiliations:** Maturity Mark Services Co., White Plains, New York, United States

## Abstract

This 26th annual symposium again showcases a world-wide musical genre in a regional setting with local performers. Texas Blues has emanated from a pantheon of talented artists beginning in the earliest days of Roots and Blues music to the present day, and Austin has become the epicenter of this talent and music. With legendary classic blues musicians from the early 2oth century to emerging younger musicians who re-energize and re-invent this uniquely American musical genre, to legendary music labels and venues like Antone’s which continue to engage blues music artists of all ages, Austin continues to be the home of Texas Blues. Lifetime Achievement Award -winning 89-year-young, Miss Lavelle White epitomizes the resilience and energy interchange of both the performer and the music, and their mutual contribution to longevity and continued engagement. Still performing after 70 years, she has influenced generations of younger prominent blues performers and continues to appear weekly at local blues mecca Antone’s along with her Grammy Award winning band and is preparing her fourth album release. This session will celebrate her music, her artistry, and her continued success as an older blues performer. A visit to a local blues venue later in the evening will allow for a true appreciation of the blues music scene in Austin.

